# Microplastics in Malaysia's Aquatic Environment: Current Overview and Future Perspectives

**DOI:** 10.1002/gch2.202300047

**Published:** 2023-07-12

**Authors:** Raja Norimie Raja Sulaiman, Aznizam Abu Bakar, Norzita Ngadi, Izzat Naim Shamsul Kahar, Abu Hassan Nordin, Muhammad Ikram, Walid Nabgan

**Affiliations:** ^1^ Faculty of Chemical and Energy Engineering Universiti Teknologi Malaysia Skudai Johor 81310 Malaysia; ^2^ Faculty of Applied Sciences Universiti Teknologi MARA (UiTM) Arau Perlis 02600 Malaysia; ^3^ Solar Cell Application Research Lab Department of Physics Government College University Lahore Lahore Punjab 54000 Pakistan; ^4^ Departament d'Enginyeria Química Universitat Rovira i Virgili Av Països Catalans 26 Tarragona 43007 Spain

**Keywords:** aquatic environment, contamination, detection, Malaysia, microplastic, occurrence, remediation

## Abstract

Microplastic pollution has adversely affected the aquatic ecosystem, living creatures, and human health. Several studies in Malaysia have provided baseline information on the existence of microplastics in surface water, ingestion by marine life and sediment. Also, humans are exposed to microplastic due to consumption of contaminated abiotic and biotic products, such as processed seafood. Nonetheless, knowledge is still scarce among Malaysian on the potential remediation and pollution management of microplastics, which poses a significant challenge to preserve a good environmental status. Green technologies also other alternative to mitigate the contamination of microplastics for sustainable future. Hence, this review aims to provide an overview of microplastic's occurrence, fate, and implications in Malaysia's aquatic environment. Detection of microplastics from the water surface, ingestion by aquatics, and sediment samples are highlighted. Available different treatment processes toward microplastic remediation are also discussed. Additionally, the potential challenges, current perspective for plastic management in Malaysia, as well as green strategies for reducing microplastic contamination are also put forward. The goal of this work is to improve the understanding of the seriousness of microplastic contamination in aquatic environments, thus encouraging key concerns that need to be investigated further.

## Graphical abstract

This review presents an overview of microplastic's occurrence, and implications in Malaysia's aquatic environment. The microplastic occurrence can be due to hydrological and environmental conditions, microplastic features, seasonal variation, population density, human activities and industrial. Available treatment processes, potential challenges, current perspective for plastic management in Malaysia, as well as green strategies for reducing microplastic contamination are put forward.

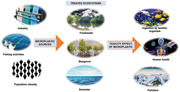

## 1 Introduction

Plastic is discharged into the environment through a number of pathways, including ordinary plastic goods, plastic degradation, industrial, wastewater treatment plants, and so forth.^[^ ^1^, ^2^ ^]^ Such condition may disturb the food chain after entering the water system that then devoured by benthic organisms, thereby providing a major health concern.^[^ ^3^, ^4^, ^5^ ^]^ Apart from apparent macroplastic, there is a concern about microplastics with a diameter of less than 5 mm.^[^ ^6^ ^]^ These microplastics are created for a certain size or result from macroscale plastic's physical, photochemical, or biological degradation into tiny fragments.^[^ ^7^ ^]^

Microplastics include primary and secondary based on the origin of these plastics. Primary microplastics include synthetic plastic pellets, beads, nurdles, fibers, powders, and pellets. Commonly, they are used as raw materials for making plastics (resin), and industrial products (clothing textiles, personal care, and cosmetic).^[^ ^8^ ^]^ Meanwhile, secondary microplastic are derived from the breakdown of larger plastic debris through gradual fragmentation or degradation via several process such as weathering, photolysis, abrasion, mechanical, and even microbial decomposition.^[^ ^9^, ^10^, ^11^, ^12^ ^]^

Weathering is an important process for the fragmentation and release of microplastics to environment. Nevertheless, the rate and extent of microplastic weathering predominantly affected by their physicochemical properties and environmental conditions.^[^ ^13^ ^]^ For instance, the weathering rate of microplastic is usually lower in water than on land. Throughout this process, both oxygen containing functional groups and specific surface area of microplastic increase as well as influencing their sorption and aggregation with other co‐existing constituents.^[^ ^14^ ^]^

On the other hand, the penetration of these microplastic into water bodies occurs via hydrodynamics and adhesion effects. Such situation leads to the uptake of the microplastic by aquatic life, hence threatening the environment.^[^ ^15^, ^16^, ^17^ ^]^ Several studies have reported the ingestion and chemical infection of several marine populations, including invertebrates (zooplankton, polychaetes, shrimps, mussel, clam, scallop and worms) and vertebrates (fish).^[^ ^18^, ^19^, ^20^, ^21^ ^]^ For instance, Hossain et al.^[^ ^22^ ^]^ reported microplastic were found in the gastrointestinal tract of two types of shrimps. Van Cauwenberghe et al.^[^ ^23^ ^]^ claimed the ingestion of microplastic in the marine invertebrates of mussel and lugworm species. Also, Cho et al.^[^ ^24^ ^]^ revealed the contamination of four invertebrate's bivalve species, including oyster, mussel, clam, and scallop. Meanwhile, Collard et al.^[^ ^25^ ^]^ also discovered the presence of 80% microplastic (124 to 438 µm) in the livers of anchovy. It can be inferred that mostly the marine invertebrates were easily contaminated by microplastic. Sfriso et al.^[^ ^26^ ^]^ believed that microplastic is strongly resembles the food, which is closely linked to the feeding mode and size of the marine organisms. Given that the particles are in the range of plankton size, marine invertebrates can consume microplastics using a variety of feeding strategies. For example, mussels (filter feeders), lugworms (deposit feeders), and sea cucumbers (detritivores) were observed to consume microplastics. Similarly, Nanthini devi et al.^[^ ^27^ ^]^ also clarified that higher scores of microplastics contamination among the smaller marine organisms is due the higher specific surface of interaction of these small organisms with the surrounding habitat.

Plastics contain many additives that can be toxic when released into aquatic system.^[^ ^28^ ^]^ The additives usually mixed with the plastic during processing and fabrication of products. Amongst the additives used are fillers (50%), plasticizers (10%–70%), flame retardants (10%–20%), colorants (1%–4%), stabilizers (0.1%–2%) and lubricants (0.5%–2%).^[^ ^29^ ^]^ These additives may slowly migrate from plastics into the environment, potentially having negative consequences on ecosystems.

The potential health hazards related to microplastic ingestion can be divided into chemical, physical particles, and microbial pathogens. In terms of chemical risk, plastic can leach estrogenic chemicals (Bisphenol A), leading to metabolic disorders in infected mammals, namely obesity, diabetes, cancer, low sperm count, and early puberty.^[^ ^30^, ^31^ ^]^ The microplastic can move over the gastrointestinal area and is released via excretion for ingestion via physical particles. However, the accumulation of plastic can occur elsewhere in the mammal body. The symptom can be detected through the physiological disorder of the gastrointestinal and disruption in the circulatory system, namely cardiac tissue, cardiovascular diseases, etc.^[^ ^31^, ^32^ ^]^ Microplastic ingestion can cause death since it can encourage gut microbiota dysbiosis in fishes, affecting the immune system.^[^ ^33^ ^]^

Development of the plastics industry in Malaysia over five decades has provided an abundance of advantages for society. According to the Malaysian Plastics Manufacturers Association (MPMA), plastic industries in Malaysia contributed RM30.98 billion to the national economy in 2018, signifying 4.7% of the country's gross domestic product (GDP).^[^ ^34^ ^]^ According to the Housing and Local Government Minister, Malaysia's residents have reached over 32 million and produced 38000 metric tonnes of waste daily. Out of this waste, only 24% was recycled, while other remaining 76% was landfilled.^[^ ^35^ ^]^

Concurrently, our country is struggling with the coronavirus (COVID‐19), which has led to the medical sector's sudden surge of plastic waste. According to Khoo et al.,^[^ ^36^ ^]^ plastic medical waste increased during the COVID‐19 pandemic since personal protective equipment (PPE), including face masks and face shield hand gloves, comes from plastic materials. Due to health and hygiene concerns, these disposable plastics have caused plastic waste generation. This is supported by Jędruchniewicz et al.^[^ ^37^ ^]^ who reported that the discarded disposable gloves could be a source and a vector of environmental pollutants during the pandemic Covid 2019. Not only that, United Nations Comtrade (UNC) data indicates that Malaysia imported 333.5 million kilos of plastic waste in 2019, not including the one imported illegally.^[^ ^38^ ^]^ Our Malaysian people already produce a lot of plastic waste ourselves, and it does not make sense for the authorities to import more such waste.

Data on the environmental behavior and toxicity of microplastics in Malaysia are scarce. Besides, knowledge about the available remediations and challenges in microplastic management remains limited. Hence, this review ultimately aims to give further comprehension on the seriousness of microplastic pollution from various regions in Malaysia as well as their detection from water surface, aquatics life and sediment. The paper also discusses the available microplastic remediation, challenges, and perspective for plastic pollution management in Malaysia, as well as green strategies for reducing microplastic contamination in water bodies.

## 2 Methodology

Literature data were obtained from databases such as science direct and web of science with keywords used for extracting and analyzing literature data including occurrence, detection, removal, challenges, and future perspective of microplastic especially in Malaysia.

## 3 Heterogeneity on Abundance and Distribution of Microplastic in Malaysia

Based on the statistical analysis, from 2016 to 2018, Malaysia's total plastic waste was generated from 2.45 to 2.65 Mt/year.^[^ ^39^ ^]^ To develop recycled products, China, Hong Kong, Malaysia, Taiwan, and India have become the top importers of plastic waste in Asia. Malaysia has been importing plastic waste at a lower rate from 2006 to 2010.^[^ ^40^ ^]^ However, this trend changed and worsened in 2018 when there was a remarkable increase in foreign plastic waste in Malaysia, thus making our country a dumpsite. Actually, this issue was triggered by the Chinese government, which banned most plastic waste imports from January 2018 agreeing to their “National Sword Policy” (NSP) to decrease pollution.^[^ ^39^, ^40^, ^41^ ^]^ Reduction of low‐quality, challenging‐to‐sort‐and‐recycle plastic imports is one goal of China's NSP. Accordingly, the solid waste trade system is anticipated to change, either by altering the location of the solid waste or by raising the standard of waste to conform to the NSP.^[^ ^42^ ^]^ This announcement has a disruptive impact on plastic waste management.^[^ ^43^, ^44^ ^]^ Also, such situation causes the top exporters such as United States and European nations started shipping their plastic waste to other Asian countries including Malaysia. Malaysia, which has become the world's largest importer of plastic waste, also involves with illegal imports by reckless importers who were transporting the rubbish without adequate documentation and using code without a permit. These illegal plastic wastes typically offer low quality, contaminated, non‐recyclable and seem to be destroyed illegally, releasing toxic substances.^[^ ^40^ ^]^

According to The Star.,^[^ ^38^ ^]^ ≈148 000 tonnes of plastic was used for food packaging in Malaysia in 2020. On average, ≈16.78 kg of plastic packaging was used by Malaysians annually. Conferring to Ellen.,^[^ ^45^ ^]^ the cost of using plastic packaging and the cost of the greenhouse gas released from its production is predicted at USD 40 billion annually, higher than the plastic packaging industry's profit pool. Based on the World Wide Fund (WWF) report, Malaysians contribute the biggest per‐capita plastic packaging users in a region responsible for more than half the plastic litter in the world's oceans.^[^ ^38^ ^]^ Furthermore, the increasing population with fast urbanization without proper plastic waste management tends to disturb important natural systems, including ocean and coastal areas, and clog the urban infrastructure.^[^ ^46^ ^]^ About 3 to 13 million tonnes of plastic waste enter the oceans annually, causing nearly 40%–50% of marine plastic pollution from single‐use consumer packaging.^[^ ^47^ ^]^ Other study reported annually ≈2.4 million tonnes of plastic waste could enter the ocean via the riverine system.^[^ ^48^ ^]^ Then, it is reported the estimated annual marine debris in Malaysia ranges from 0.14 −0.37 million metric tons/year.^[^ ^49^ ^]^

On the other hand, Malaysia is ranked as the eighth‐highest contributor to marine plastic pollution, whose 60% of plastic debris entering the ocean.^[^ ^47^, ^50^ ^]^ Eastern states of peninsular Malaysia, mainly Terengganu, Kelantan, and Pahang, contribute much plastic waste, generating 0.71 kg/capita/day.^[^ ^51^ ^]^ Certain activities, namely agricultural, industrial, tourism, fishing, and urban areas are the main factors contributing to plastic waste pollution.^[^ ^52^ ^]^ Application of biosolids and compost, wastewater irrigation, mulching film, polymer‐based fertilizers, and atmospheric deposition are the main sources of microplastic pollution in agricultural soils.^[^ ^53^ ^]^ Besides, Jang et al.^[^ ^54^ ^]^ claimed that human activity highly affects the abundance contamination of microplastics in the environment. For instance, polystyrene (PS) was more abundant in the aquafarm site, reflecting more PS aquaculture buoys. Meanwhile, polypropylene (PP) was more abundant at the rural site due to the high use of PP ropes and nets during fishing activity.

Observation by Fauziah et al.^[^ ^55^ ^]^ on the Malaysian beaches have provided a clearer understanding of the distribution of plastic debris. A total of 265.30 gm^−2^ of small plastic debris was collected from several beaches, with the highest number from Seberang Takir Beach (879 particles m^−2^), followed by Batu Burok Beach (780 particles m^−2^). Plastic line, foam, and film were predominantly found in fishing beach areas while film, foam, and fragment were found in recreational beach. It can be inferred that the abundance of different type plastic waste is vary depending on the functions of the beach. Besides, climate also influence the presence of the plastic debris wherein ≈86.64% of total debris items was reported during southwest monsoon at Sabah.^[^ ^16^ ^]^

Malaysia contributes ≈0.199 trillion microplastics from personal care and cosmetic products toward marine ecosystem.^[^ ^56^ ^]^ These microplastic have been ingested by several marine species in Malaysia water bodies such as *Scapharca cornea*, cage cultured and wild *Lates calcarifer*, *Namalycastis. Sp*, and zooplankton.^[^ ^57^, ^58^, ^59^, ^60^ ^]^ It is revealed that plastic debris has been found in the fish tissue.^[^ ^61^, ^62^ ^]^ Not only that, microplastics also found in the human colon.^[^ ^5^ ^]^ Amongst type of microplastic polymers found are PP and polyethylene terephthalate (PET).^[^ ^63^, ^64^ ^]^ Other hazard organic contaminants, namely polyamide (PA), polyester (PES), polymerizing vinyl chloride, and acrylics in microplastics were reported as well.^[^ ^65^ ^]^ Microplastics are omnipresent with long residence time, high stability, easily fragmented, and capable of absorbing other pollutants.^[^ ^66^, ^67^, ^68^ ^]^ Thus, seafood intake could be a detrimental exposure of microplastic to Malaysians since they are essential part of the dietary sources.

### 3.1 Microplastic Ingested by Benthic Organism in Malaysia

Microplastics turns out to be a hot subject of study amongst researchers worldwide. Few studies on the ingestion of microplastics via numerous types of benthic organisms in several Malaysian water areas, especially in eastern Peninsular Malaysia, mainly in Terengganu and Pahang, have been reported (**Table** **1**). Setiu Wetland in Terengganu comprises several interconnected ecologies, namely seashore, ocean, mudflat, waterway, cove, river mouth, island, and coastal forest, filled with various types of flora and fauna living in it.^[^ ^69^ ^]^

**Table 1:** Summary of reported microplastic of various regions in Malaysia

Location/Ecosystem	Sample	Microplastic abundance	Characteristic of microplastic	References
Setiu Wetlands/ Seawater	Ingestion by benthic organisms	0.00090547 particles m^−3^ (0.12 −9.5 mm)	Filaments, transparent, PE and PA	[57]
		0.00007496 particles m^−3^ (<1 mm)	Fiber, fragment threadlike Black, transparent, blue PA, and polyvinyl alcohol (PVA)	[58]
		0.00020502 particles m^−3^ (0.9‐4.7 mm)	Filaments, transparent PP, and PA	[59]
Terengganu Coastal Waters, Southern South China Sea/ Seawater	Ingestion by benthic organism	0.03‐2.04 particles m^−3^ (0.02‐ 1.68 mm)	Fragment, fiber, PA	[74]
Terengganu estuary and offshore waters, Malaysia/ Seawater	Ingestion by benthic organism	291‐812 particles m^−3^ (96.8‐361.7 µm)	Offshore: fiber; Estuaries: fiber PP, PE, and PA	[60]
Tanjung Penyabung, Mersing and Pantai Remis, Johor/ Seawater	Ingestion by benthic organism	5.17‐9.88 particles m^−3^); (0.063‐5 mm)	Black, blue, PE, PP, acrylonitrile butadiene styrene (ABS), PS and (PET)	[77]
Pulau Pangkor, Perak, Malaysia/ Seawater	Ingestion by benthic organism	0.000015 particles m^−3^ (0.5 to 2 µm)	Fiber, black, PE and poly (methyl methacrylate) (PMMA)	[81]
Klang River estuary, Malaysia/Freshwater	Ingestion by benthic organism	0.0000005 −0.00000175 particles m^−3^ (300‐1000 µm)	Fiber, black, polyethylene‐propylene‐diene (PE‐PPD) and PES	[82]
Terengganu Coastal Waters, Southern South China Sea/ Seawater	Surface water	0.00090547 particles m^−3^ (0.02‐ 1.68 mm)	Fragment, fiber, PA	[74]
Terengganu estuary and offshore waters, Malays/Seawater	Surface water	211.2‐ 421.8 particles m^−3^ (96.8‐361.7 µm)	Offshore: fiber, fragment, and pellets	[60]
Setiu Wetlands/ Seawater	Surface water	0.00036 particles m^−3^ (200 µm)	Transparent, film, and filament	[83]
Sungai Dungun, Terengganu/Freshwater	Surface water	0.1771 particles m^−3^ (200 µm)	Fiber, fragment, black and transparent, PP, polyacrylonitrile(PAN) and rayon	[87]
Cherating river and Cherating mangrove, Pahang, Malaysia/Freshwater	Surface water	0.0051‐0.0070 particles/m^3^ (0.5 to 1.0 mm)	Fragment, white color	[88]
Kuala Nerus, Terengganu and Kuantan, Pahang/Freshwater	Surface water	0.00013‐ 0.00069 particles m^−3^ (200 µm)	Fragment, Filament, Irregular Black Grey, PA and PP, PES, PS	[89]
Skudai and Tebrau Rivers/Freshwater	Surface water	0.20‐0.68 particles m^−3^ (1000 to 5000 µm)	Not mentioned	[90]
Bangi, Selangor/Freshwater	Surface water	17 particles m^−3^ (200‐500 µm)	Fragment, film, pellet, foam Polymer: PE	[91]
Setiu Wetlands/ Seawater	Sediment	0.00000597 particles m^−3^(200 µm)	Transparent, film, and filament	[83]
Terengganu Coastal, Malaysia/ Seawater	Sediment	0.00304‐0.00306 particles m^−3^ (>125 µm)	Fiber, black Seasonal	[92]
Santubong and Trombol beaches, Kuching/beach/ Seawater	Sediment	0.0000000358‐0.0000017343 particles m^−3^ (1‐5 mm)	Fragment, PP, and PET	[93]
Baram River, Sarawak Malaysia/ Freshwater	Sediment	0.0000005188‐ 0.00000087particles m^−3^ (0.3‐1 mm)	Fragment, blue, PE, PET fibres, silicon polymer	[105]
Sementa Mangrove, Kapar, Selangor/mangrove/ Freshwater	Sediment	0.418 particles m^−3^ (1‐5 mm)	PS foam and plastic fragments	[95]

*Scapharca cornea*, a bivalve invertebrate, is widely distributed in Setiu Wetland. Ibrahim et al.^[^ ^57^ ^]^ have reported the ingestion of microplastics in *Scapharca cornea.sp*. wherein ≈0.00090547 particles m^−3^ of microplastics (0.12 to 9.5 mm) have been successfully detected in 120 handpicked wild *Scapharca cornea*.sp. Other than that, Ibrahim et al.^[^ ^58^ ^]^ claimed no studies documenting the ingestion of microplastics by Malaysian estuary fish. Throughout their study, the comparison of microplastic ingestion between two fishes derived from different habitat locations was reported. The finding revealed the presence of the total 0.00007496 particles m^−3^ microplastics in estuarine fish (wild and cage‐cultured) of *Lates calcarifer*. Microplastics are more prevalent in wild species than in cage‐cultured species, which is related to habitat dynamics and feeding behavior. Even though many benthic creatures have consumed microplastics, scientific evidence on consumption of microplastics is still lacking concerning deposit‐feeding *polychaete Namalycastis.sp* or bait worm that live estuarine environments. Then, the ingestion of microplastics by *polychaete Namalycastis .sp* (0.00020502 particles m^−3^) was conducted as well.^[^ ^59^ ^]^

It can be inferred that most studies conducted in Setiu Wetland involve the ingestion of microplastic among marine invertebrates. Some studies found that these invertebrates tend to be selected as ideal test organisms since they can be easily found, simple to sample, stress‐resistant, sessile animals and most crucially are popularly known as benthic filter feeder for microplastics.^[^ ^70^, ^71^ ^]^ This is supported by Parsaeimehr et al.^[^ ^72^ ^]^ who discovered that small animals like invertebrates commonly consume toxins like microplastics, which they can absorb. The presence of microplastics in marine species is important for the estuarine food web since the study can serve as a baseline for these microplastics to go up the trophic chain.^[^ ^73^ ^]^

Meanwhile, becoming a feeding ground for zooplankton, the widespread accumulation and distribution of microplastics at the sea surface is worrying. Research on the contamination of microplastic in the seawater and zooplankton has been carried out in the Terengganu coastal area. Microplastic ingestion was obtained from different clusters of zooplankton that are 2.04, 1.71, 0.63, 0.60, 0.24, and 0.03 particles m^−3^ for cyclopoids, calanoids, fish larvae, polychaetes, shrimp and zoea and chaetognaths, respectively.^[^ ^74^ ^]^ Similar method was used by Taha et al.^[^ ^60^ ^]^ to investigate the ingestion of microplastics in seawater and zooplankton from Malaysia's Terengganu Estuary to offshore waters. Results show that 812 and 291 particles m^−3^ of microplastic have been successfully extracted from estuaries and offshore, respectively. Throughout these studies, it is demonstrated that zooplankton also plays a role as a repository for microplastic in the marine ecosystem. Despite its small size, zooplankton is an important component of the pelagic ecosystem since it serves as a link between primary producers and higher trophic levels, such as humans.^[^ ^75^ ^]^ Zooplankton may act as a pathway for microplastics to infiltrate the food chain through ingestion, hence endangering secondary producers, apex predators, and possibly even human health.^[^ ^76^ ^]^

The widespread presence of microplastics in commercial marine fishes from Malaysian oceans poses a risk of human exposure through fish consumption. This is supported by Jaafar et al.^[^ ^77^ ^]^ who claimed the contamination microplastics occurred in the gastrointestinal tract area (GIT) and gills of 158 fish from 16 species collected from two sites in Malaysian coastal waters. About 86, and 92% of microplastic have been detected in the GIT and gills of the studied fish, respectively. Zhu et al.^[^ ^78^ ^]^ believed the smaller plastics predominated in GIT since fish mistaking them for food whereas the larger one highly concentrated in gills, indicating that they were less likely to be flushed back into the sea. Other studies also stated that the high occurrence in fish gills showed that the uptake of microplastics via ventilation is a main pathway of microplastics ingestion in fish.^[^ ^79^ ^]^

Other than that, it is found that about 9.88 and 5.17 particles m^−3^ of microplastic were obtained from urban and less urbanized areas, respectively.^[^ ^77^ ^]^ Such high incidence of microplastics in urban areas may be related to the densely populated and visited by local people and tourists.^[^ ^80^ ^]^ Other popular area studied is Pulau Pangkor with an increasing number of visitors and development, making it excellent for long‐term research. Husin et al.^[^ ^81^ ^]^ examined samples of *S. horrens* from Pulau Pangkor, which were dissected, and their intestines were digested. Result showed that ≈0.000015 particles m^−3^ microplastic was found in *S. horrens*. Then, Klang River estuary is an important ecosystem that receives various contaminants from urbanized, highly populated areas and the busiest maritime center in Selangor, Malaysia. Using this place as a research study, Zaki et al.^[^ ^82^ ^]^ reported microplastics were found in concentrations ranging from 0.0000005 −0.00000175 particles m^−3^ in the gastropods from a variety of land use regions such as ports, residential, and industrial areas. It can be concluded that mostly the studies showed high occurrence of microplastic in urban areas compared to the rural areas. According to Mihai et al.,^[^ ^80^ ^]^ it is still unclear how much rural areas contribute to global plastic pollution since there are few data on rural waste at the national level.

### 3.2 Microplastic from Surface Water in Malaysia

Pieces of evidence on the microplastic abundance from Malaysian surface waters are tabulated in Table 1. Md Amin et al.^[^ ^74^ ^]^ reported that 0.00090547 particles m^−3^ were discovered with an average abundance of 0.0033 particles m^−3^ in surface seawater. Taha et al.^[^ ^60^ ^]^ carried out the first study focusing on the microplastic pollution in surface water of estuaries and offshore in Malaysia. Results revealed that Terengganu Estuary has a greater microplastic density (421.8 particles m^−3^) than offshore waters (211.2 particles m^−3^). Meanwhile, Ibrahim et al.^[^ ^83^ ^]^ have reported the distribution of microplastics in surface water in the South and North Setiu Wetland in the South China Sea. Their findings indicated the presence of a total of 0.0036 particles m^−3^ of microplastics from surface water. As for the surface water of marine ecosystem, it is observed there are high occurrence of microplastic in estuarine compared to the coastal and Setiu wetland areas. This can be related to the exposure of estuarine to high prevalence of microplastics sources such as wastewater treatment, industry, agriculture, and urban areas.^[^ ^11^, ^84^ ^]^ Besides, tropical cyclones, floods, erosion processes, and changing water flow and levels affected estuarine environments, hence releasing of pollutants in estuaries.^[^ ^85^ ^]^

Although significant research efforts have focused on the effects of microplastic on the marine ecosystem, it is evident that inland water is experiencing the same issues. Rivers are possible water supplies and routes for microplastic to the oceans, mostly because of a terrestrial based activity.^[^ ^86^ ^]^ Several studies have reported the contamination of the microplastic from surface water of the freshwater ecosystem. For instance, Hwi et al.^[^ ^87^ ^]^ have investigated the occurrence of microplastic in Sungai Dungun, Terengganu with the range concentration of microplastic obtained was 0.1771 particles m^−3^. The presence of metals released from microplastic such as stanum, arsenic, manganese, zinc, copper, iron, and aluminum, was also observed. Besides, Pariatamby et al.^[^ ^88^ ^]^ have reported the presence of microplastics in surface water samples obtained from the Cherating river and Cherating mangrove in Pahang. The midstream region provided the highest microplastics abundance, with an average abundance of 0.0070 ± 0.0033 particles m^−3^, followed by the mangrove (0.0051 ± 0.0053 particles m^−3^). Khalik et al.^[^ ^89^ ^]^ conducted a field study on microplastics analysis in Malaysian marine waters at Kuala Nerus, Terengganu, and Kuantan, Pahang. It was found that the total microplastic particles (minimum 5 mm) found in Kuala Nerus and Kuantan port were 0.00013‐0.00069 and 0.00014‐0.00015 particles m^−3^, respectively. Additionally, Sarijan et al.^[^ ^90^ ^]^ have reported an abundance of microplastic between the Skudai and Tebrau Rivers. It is observed that the average concentrations of particles found in the Skudai and Tebrau Rivers were 0.20 and 0.68 particles m^−3^, respectively that were dominated by size ranging from 1000 to 5000 µm. Then, Suardy et al.^[^ ^91^ ^]^ also claimed the presence of 17 particles m^−3^ microplastics that come from personal care from surface water of Sungai Langat and Tasik Cempaka in Bangi, Selangor. All these studies provided new insights on the microplastic pollutions and their impact on Malaysia's freshwater systems.

### 3.3 Microplastic from Sediment in Malaysia

Ibrahim et al.^[^ ^83^ ^]^ have reported the distribution of microplastics in estuarine sediments in the South and North Setiu Wetland in the South China Sea. Their findings indicated the presence of a total of 0.00000597 particles m^−3^ of microplastics from dry sediment. On the other hand, there is a scarcity of information on the amount of microplastic found on Southeast Asian Sea turtle breeding beaches. Between October and November 2018, Hamza et al.^[^ ^92^ ^]^ have collected samples from four sea turtle nesting beaches in Terengganu, Malaysia to evaluate microplastic abundance. They have successfully found about 0.00304 to 0.003058 particles m^−3^ microplastic objects using optical observation. The microplastic found in sea turtle were divided into four categories: fibers, fragment, foam, and films. The most typical shape was that of fibers (96.18%). Black was the most common color discovered (35.64%), followed by transparent (24.53%).

Besides, Noik and Tuah^[^ ^93^ ^]^ have surveyed the abundance of plastic at two sandy beaches in Kuching, Sarawak, Malaysia, Santubong and Trombol. Results revealed that Santubong and Trombol provided a mean weight of 0.0000000358 and 0.0000017343 particles m^−3^, respectively. Both places are situated at an estuary, hence providing the high possibility of translocating plastics from other inhabited zones. Meanwhile, Baram River is one of Sarawak's greatest rivers, with many large industries along its banks, including plywood, sawmills, shipyards, interisland ports, and other wood‐based industries. Study revealed that about 0.0000005188 to 0.00000087 particles m^−3^ was discovered in the sediment of Baram River.^[^ ^94^ ^]^

Emphasizing areas isolated from anthropogenic activities, Barasarathi et al.^[^ ^95^ ^]^ as quantified a significant total quantity of 0.418 particles m^−3^ microplastic in a mangrove forest in Kapar, Selangor, Malaysia. It is revealed that the most pronounced microplastic found is fragment type that is not only limited to the surface but has penetrated deep into the soil core. Their presence may be due to consumer use of things such as bottles, plates, toys, etc. Hence, it is vital to have countermeasures to keep Malaysia's mangrove forests from microplastic contamination.

### 3.4 Microplastic Characteristics in Malaysia

**Figure 1:**
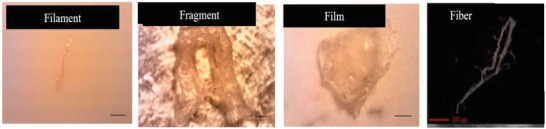
Images of several common shapes of microplastic. Reproduced with permission.^[^ ^59^ ^]^ Copyright 2021, Elsevier.

Table 1 also exhibits the several characteristics of microplastic found in Malaysian water bodies. Size of microplastic has a greater impact on the organisms since the interaction of microplastic on the organism increases with the reduction of plastic's size.^[^ ^59^, ^77^, ^96^ ^]^ For instance, plastic with size < 1 mm is able to penetrate the cell barrier, hence adversely causing the oxidative damage, fecundity, and immunological responses in the organism.^[^ ^97^, ^98^ ^]^ Microplastic also can be sorted into several diverse shapes as shown in **Figure** **1**, namely fiber, film, filament, fragment and irregular.^[^ ^59^ ^]^ Meanwhile, identifying the type of microplastic polymer is crucial since it determines its chemical composition.^[^ ^58^, ^99^, ^100^ ^]^ Density separation offers potential information in analyzing the chemical components making up the microplastic.^[^ ^58^ ^]^ Polymers such as PE and PP provide density lower than water while the polymers, including PS, PES, and PA are slightly dense than water.^[^ ^101^ ^]^ Besides, colors of microplastic such as transparent, red, blue, green, black, brown, and others represent their different specific polymers.^[^ ^101^, ^102^ ^, 103]^ It is reported that a fragment microplastic in blue or green color was identified as PE.^[^ ^104^ ^]^ However, some studies specified that the color and the chemical composition of the plastics have no correlations.^[^ ^57^ ^]^

**Figure 2:**
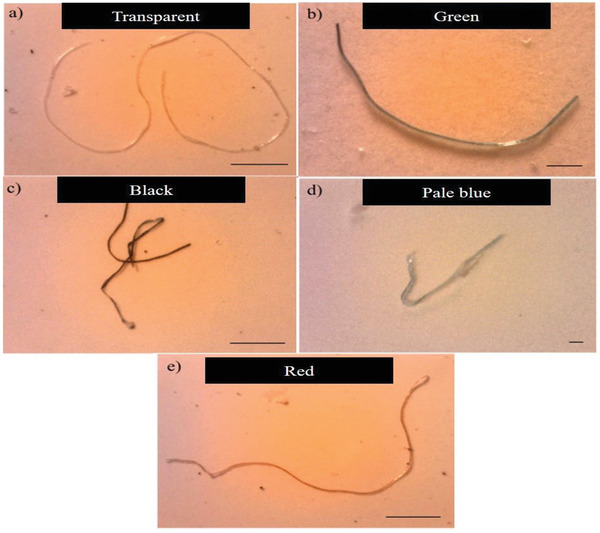
Images of several common colors of microplastic. Reproduced with permission.^[^ ^58^ ^]^ Copyright 2017, Malaysian Analytical Sciences Society.

Majority of the microplastics discovered in Setiu Wetlands area were filaments (0.12 to 9.5 mm) and exist in a variety of colors, including translucent, blue, green, white, black, orange, red, purple, grey, and brown.^[^ ^57^ ^]^ Furthermore, the presence of microplastics (<1 mm) in black threadlike, transparent and blue threadlike shapes of wild and cage‐cultured *Lates calcarifer* was successfully reported as shown in **Figure** **2**.^[^ ^58^ ^]^ Similarly, research by Hamzah et al.^[^ ^59^ ^]^ reported that the microplastic found in the ingested polychaete, *Namalycastis. Sp* in a size range of 0.9 – 4.7 mm from the Setiu Wetland's estuary ecosystem was highly dominated by filaments (99.79%). Meanwhile, the transparent color of microplastic provided the highest percentage (84.71%) compared to the other colors. Such microplastics with filament shape and transparent color seem like the structure of rotten Nypa (natural food), hence was mistakenly taken as a food source by *Namalycastis* sp. This is in accordance with Li et al.,^[^ ^104^ ^]^ who indicated that the misidentification of microplastic colors raises the probability of a food source during microplastic ingestion.

Terengganu coastal and estuary also has become a hotspot for microplastic pollution. Fiber and fragments are highly dominated by the shape of microplastic found in that region.^[^ ^60^, ^74^, ^92^ ^]^ This is strongly supported by Md Amin et al.^[^ ^74^ ^]^ who reported the fiber and fragment microplastic (0.02–1.68 mm) found from ingestion of zooplankton. Meanwhile, referring to the case study from Taha et al.^[^ ^60^ ^]^ three types of microplastics were found in the surface water from Terengganu estuary to offshore waters, namely fibers, fragments, and pellets. In detail, from the estuary area, microplastic (96.8 µm) were dominated with fiber (73.8%), fragments (22%) and pellets (4.2%). Similarly, from the offshore area, microplastic (361.7 µm) with fiber are dominant with 80.8%, fragments (18.6%) and pellets (0.17%). Furthermore, it was shown that zooplankton included two forms of microplastic that are fibers and fragments. Specifically, total ingested microplastics by zooplankton offshore covered 94% and 6% of fibers and fragments, respectively.

Other than that, ≈78% and 22% of fibers and fragments were obtained from zooplankton in the Terengganu estuary, respectively. It is noted that the size of ingested microplastics offshore is more significant compared to the estuary. According to Jaafar et al.,^[^ ^77^ ^]^ the isolated microplastic (0.063–5 mm) from the studied fish is made up of fibers (80.2%), fragments (17.7%), and filaments (3.1%). Meanwhile, PE, PP, ABS, PS, and PET were discovered in the selected microplastic using Fourier transform infrared (FTIR) and Raman spectroscopy (RS).

Based on the field study by Khalik et al.,^[^ ^89^ ^]^ it is revealed that Kuala Nerus contributed ≈76.2% and 23.76% of microplastic with fragment and filament shapes, respectively. Meanwhile, the microplastic with fragment shape constituted 50.8%–66.1% of the total microplastic for Kuantan samples. Likewise, microplastic with filaments and irregular contributed to the range of 20.9%–38.3% and 10.8%–19.1%, respectively. In terms of colors, there were eight different colors found in microplastic, namely black, blue, brown, grey, red, orange, yellow and transparent. However, the microplastic particles were dominated by black (65.5%) in Kuala Nerus, and grey color is dominant (48.7%) in Kuantan.

As stated by Sarijan et al.,^[^ ^90^ ^]^ microplastic in the film shape is highly found in both Skudai and Tebrau River, Johor. Meanwhile, yellow, and white colors of microplastic were dominant in the Skudai River, and blue appears to be the dominant color found in Tebrau River. Furthermore, the work of Noik and Tuah^[^ ^93^ ^]^ revealed physical characteristics of plastic fragments and debris in Santubong and Trombol; Kuching was primarily in fragment shape. PP and PE were the most common plastic polymers found on both beaches, according to FTIR fingerprinting study. Fibers (91%) with a black color were the most common microplastics discovered in gastropods (50%) at Klang estuary. The major polymer components were PE‐PPD and PES. Assessing the microplastic contamination of gastropods gives insight into the feasibility of using gastropods as bioindicators for monitoring and baseline investigations.^[^ ^82^ ^]^

Microplastics were found in fragments with a size of 0.5 to 1.0 mm in surface water samples taken from the Cherating river and the Cherating mangrove in Pahang, and white‐colored microplastics were common.^[^ ^88^ ^]^ From the evaluation of microplastic in Pulau Pangkor, Husin et al.^[^ ^81^ ^]^ have found ingested microplastic (0.5 to 2 µm) by sea cucumber, *Stichopus horrens* were commonly fiber (90%), and black (59%). PE and PMMA are two polymers that have been identified as well.

Based on the observation of microplastic in Baram River estuary, Choong et al.^[^ ^105^ ^]^ have concluded that microplastic fragments accounted for 67.8% of the total microplastics identified in the water and sediment, followed by fiber, film, pellet, and foam. PE, PES fibers, silicon polymer, nitrile, and PS were among the microplastic polymer types discovered. The most prevalent size range for microplastics in the Baram River was 0.3–1 mm, with blue being the most common color. According to Praveena et al.,^[^ ^106^ ^]^ polyester, nylon, and acrylic microplastic fiber and fragment forms with an average length of 2258.59 m were also found in these laundry water samples. In terms of polymer characteristics, PP PE, and PA are the main polymer types for microplastic found in the Setiu Wetland's estuary ecosystem. Both polyamide (PA) and PVA were the main polymers found in these microplastics, which are slightly denser than water. The high number of PA from the microplastic ingested from the cage cultured species was believed to come from the cage utilized for fish aquaculture fabricated from synthetic resources. Besides, PVA comes from the settlement of the fishing gear or nets on the bottom floor.^[^ ^58^ ^]^

Typically, PE and PP are commonly connected with food storage, consumer goods, and single‐use items, while PA is mostly employed in fishing nets and the automotive industry.^[^ ^107^ ^]^ PA are largely used for nylon substances, cloths and fishing gear, including nets and ropes.^[^ ^108^ ^]^ As the sampling location is busy with fisherman activities, the chances for the fishing gear or nets to be abandoned in the waterways are higher. Results of µFTIR spectra on the microplastic sampled from the offshore waters with fiber types were identified as PA, PE and PP representing various activities including restaurants, tourism, fishing, seafood processing industries, and marine traffic, which is performed in that area. As for the polymer characteristics, PA and PP materials are the main polymer group found in Kuala Nerus. Meanwhile, the main polymer group identified in Kuantan is PES, PS, PA, PVC, and PE. Commercial fishing and tourism are the major activities in Kuala Nerus, while Kuantan Port is one of Malaysia's big multi‐cargo ports.^[^ ^89^ ^]^

Suardy et al.^[^ ^91^ ^]^ have observed the occurrence of primary microplastic, which include microbeads in personal care products in Bangi, Selangor. It is found that the microplastic comes with a size ranging from 200–500 µm, and polymer characteristics are PE and PS. The extracted microplastic comes from various colors: white, purple, pink, brown, colorless, while the shapes were spherical, granular, and irregular.

### 3.5 Cause Analysis of Microplastic Abundance in Malaysia

Several factors influencing the abundance and distribution of microparticles in Malaysia namely hydrological conditions of water system, environmental conditions, sampling location, microplastic features, seasonal variation, population density, human activities, and industrial and domestic waste. Ibrahim et al.^[^ ^57^ ^]^ observed that the quantifications of microplastics collected from Setiu Wetlands are decreasing tremendously from station 1 (N 05°41″144″, E 102°42″629‴) to station 2 (N 05°41″423‴, E 102°42″258‴) due to the tides, winds or water movement phenomena. Station 1 provides adequate food for *S. cornea* with less water movement, providing a long duration for the microplastic to be retained there. Conversely, station 2 is entirely unsuitable due to the fast water movement from the incoming tides. Meanwhile, Ibrahim et al.^[^ ^58^ ^]^ observed that wild *L. calcarifer* had ingested a considerably higher abundance of microplastics compared to cage‐cultured *L. calcarifer*. The wild *L. Calcarifer* being positioned near the passage of the South China Sea that is rich with the microplastic particle transferred from the sea over ocean current and wind. Besides, cage‐cultured species were taken northwards from Setiu Wetlands, next to the settlement area. The cage‐cultured fishes ingested more threadlike particles compared to wild species of *L. calcarifer*, probably resulting from the disintegration of nets used in the aquaculture ponds. However, the fragment type was reported remarkably higher in quantity for wild fishes, as it could be denser than water, expected to sink and therefore being accidentally ingested by wild *L. calcarifer*.

Likewise, Hamzah et al.^[^ ^59^ ^]^ found that the high ingestion of microplastics occurred at Station 3 (ST3) (N 05^◦^40′ 54.46′′, E 102^◦^42′ 33.25′′) (692 pcs) and Station 4 (ST4) (N 05^◦^39′49.27′′, E 102^◦^43′ 57.69′’) (674 pcs), which is due to the influx from the open sea and aquaculture. It is also reported that ST2 (N 05^◦^41′30.77′′, E 102^◦^41′54.98′′) provided the highest number of individual microplastic ingestion since this area was noticeably smaller than the other stations. In terms of microplastic features, the abundance of microplastic filaments is transparent in color are misunderstood by estuarine polychaetes because the structure closely mimics the structure of their natural feed, decaying nypa. Similar observation was reported by Barasarathi et al.^[^ ^95^ ^]^ who indicated that these particles seem to be like phytoplankton, thus the tendency of being ingested as food by marine organisms becomes high. Distinctive in the population of benthic organisms in certain areas also influences the variance in microplastic ingestion from the sample species studied. The inconsistent microplastic ingestion can explain this in zooplankton in Terengganu coastal, which depends on the zooplankton abundance at different stations.^[^ ^74^ ^]^ A similar report was made by Taha et al.,^[^ ^60^ ^]^ who claimed that the number of microplastic ingested from offshore and estuaries waters is predominantly affected by the density of the zooplankton. As observed, in the offshore water area, station B3 (N 5.775, E 103.62167) provided the highest number of ingested microplastic (535 particles m^−3^) with the highest density of individual zooplankton (291.2 particles m^−3^). Conversely, station A5 (N 5.52389, E 104.25944) provided the lowest ingested microplastic (93 particles m^−3^) due to the lowest density of zooplankton (50.6 particles m^−3^). Similarly, in the Terengganu Estuary, the greatest ingested microplastic occurred in ST1 (N 5.3434, E 103.14873) (195 particles m^−3^).) with the greatest zooplankton total density (812.5 particle m^−3^). In contrast, the lowest ingested microplastic occurred in ST6 (N 5.32416, E 103.11726) (63 pcs) with the lowest total density of zooplankton (262.5 particles m^−3^).

Khalik et al.^[^ ^89^ ^]^ exhibited a high number of microplastic found in the non‐urban area (Kuala Nerus) compared to the urban area (Kuantan). Additionally, there is no significant difference between the station studied and the number of microplastic ingested in Kuantan port, showing the uniform distribution in the aquatic debris in the east coast region. This study seems to contradict with the work by Jaafar et al.^[^ ^77^ ^]^ who observed the high occurrence of microplastic were identified in the benthic organisms from the region nearby to the city region (9.88 particles m^−3^) compared to the microplastic obtained in the benthic organisms from less urbanized region (5.17 particles m^−3^). Several anthropogenic activities namely fisheries, aquaculture, agricultural, restaurants, tourism, fishing, recreation, sand mining and so on around this area, are often pointed out as essential contributors to microplastic pollution in the Setiu wetland. The main sources of microplastic pollution are believed to be fishing and tourism activities that could result in a high concentration of transparent microplastics in the marine environment. Furthermore, since plastic fibers were significant raw materials for fishing nets and lines, the availability of fiber may be related to the widespread use of fishing equipment. Monofilament fragmentation (single fiber) from fishing nets, ropes, synthetic cloth, or garment fibers determines the fiber type.^[^ ^81^, ^82^, ^88^, ^105^ ^]^

On the other hand, Noik and Tuah^[^ ^93^ ^]^ claimed that most of the plastic particles found on the beaches of Santubong and Trombol (Kuching, Sarawak) are not associated with heavy industrial goods but rather with domestic items. Broken kitchen and bathroom furnishings, motor oil containers, personal care and toiletries, children's toys, and abandoned fishing gear and storage boxes are among the items found. Besides, the unpleasant look of plastic being disseminated and deposited in the environment is often ascribed to human factors. Most of the plastic was carried a long distance by wind, water, and animals. The most prevalent storage sites for these man‐made polymers include seas, ocean gyres, islands, beaches, and rivers. Research by Hwi et al.^[^ ^87^ ^]^ discovered that the high abundance of microplastic at ST3 (N 4°46′38.8″, E 103°23′42.7″) is due to the sampling site located near the outlet of receiving waters. These riverine of Sungai Dungun filled with the domestic discharge from residential areas including Pengkalan Macang, Nibung, Sungai Buaya, Padang Jambu, and Tanjung Batu Villages.

## 4 Detection of Microplastics from Water Surface, Ingestion by Aquatics and Sediment

Filtering, which uses sieves with varying pore sizes or mesh sizes, is a popular approach for removing microplastics from water samples. When the pore or mesh size is smaller, more microplastics are detected. Khalik et al.^[^ ^89^ ^]^ used filtration with 20 µm pore size to trap microplastic from surface water samples collected using a 5.7 L calibrated steel sampler. Throughout this study, the authors did not mention specifically the size of microplastics obtained but stated only particles with a diameter of at least 5 mm were considered. Hwi et al.^[^ ^87^ ^]^ have filtered the collected 400 L surface water sample containing microplastic using sieve made of a 200 mm diameter stainless steel with a 200 µm mesh size. Digestion with hydrogen peroxide (H_2_O_2_) is a typical method for removing organic materials from water samples while preserving the tested polymer. These samples were treated with 30% H_2_O_2_ for 24 h at room temperature to oxidize organic materials. Microplastic with sizes of 1.22–1.30 mm was commonly found in their works. Meanwhile, Choong et al.^[^ ^94^ ^]^ have sieved 10 L water samples containing microplastic collected in stainless steel buckets with mesh size (0.3–5 mm) to trap the microplastics. The smallest particle (0.3–1 mm) was predominant that accounted for 36.8% of the total microplastic particles in the water samples, followed by 1–2 mm (25.7%), 2–3 mm (18.6%), 3–4 mm (11.6%), and 4–5 mm (7.4%). Pariatamby et al.^[^ ^88^ ^]^ have used a set of Tyler sieves with mesh size from 0.1 to 5 mm. It is found that microplastics of size fractions 0.5 to 1.0 mm were the most prominent up to 50% in several studied stations. The high abundance of microplastic with size>0.5 mm assumes that smaller microplastic size classes eventually sink into deeper segments, owing to the high surface‐to‐volume ratio, which favors biofouling. As can be observed, these studies used the size at least 5 mm as benchmark for the size of microplastic detected.

On the other hand, acid and alkaline digestions are more typically used to treat microplastics in aquatic species in Malaysia area. For instance, the tissue of *Scapharca cornea* was digested using 10 M sodium hydroxide (NaOH) at 60 °C and being digested 90% after 24 h of treatment. A 500 µm filter was used twice to filter the residue thereafter.^[^ ^57^ ^]^ Similarly, Ibrahim et al.^[^ ^58^ ^]^ have used 10 M of NaOH to digest the gastrointestinal tract of the cage‐cultured and wild *L. Calcarifer*. To increase the digestion, the sample was heated under temperature of 60 °C and shaken constantly at 130 rpm during the incubation period. However, the filtration of sample was carried out after 21 days. Other study by Hamzah et al.^[^ ^99^ ^]^ also reported the use of 10 M NaOH to digest specimen of *Polychaete Namalycastis* sp. The sample was wrapped in aluminium foil and placed in a 60 °C oscillation water bath until the contents were entirely digested. The digestion procedure took a maximum of 48 h to complete, depending on the size of each sample.

However, the researchers should pay attention in terms of the concentration of digestion solution for microplastic. Sun et al.^[^ ^109^ ^]^ claimed that the microplastics will be affected by the strong oxidising acid, which will degrade polymers that are sensitive to low pH. Besides, Li et al.^[^ ^110^ ^]^ reported that the effectiveness of microplastics extraction for 5 M nitric acid (HNO_3_ ) and 5 M hydrochloric acid (HCl) is lower than that 1 M HNO_3_ and 1 M HCl since PA and PE are not acid resistant, thus impacting on the overall microplastics extraction efficiency. As a result, the acidic and alkaline solution concentrations utilised should be adequate. In future trials, the balance between reagent concentration and digestion efficiency should be considered.

Density separation, which is based on the density differential between plastic and sediment particles, is another essential approach for extracting microplastics from sediments. Hamza et al.^[^ ^92^ ^]^ have separated plastic fragments with bulk of the sand using fluidized density separation system with sodium chloride (NaCI) (1.2 g cm^3^) as reagent. Sand and other particles with a higher density than the solution will settle at the bottom of the column, while those with a lower density will float to the top. Meanwhile Noik and Tuah^[^ ^93^ ^]^ reported the extraction of microplastic from sediment wherein less dense particles were separated using flotation process. Similarly, Barasarathi et al.^[^ ^95^ ^]^ used the same method with a slight modification. The concentrated saline solution was added to the sediment before settling process. Choong et al.^[^ ^105^ ^]^ have treated the sediment containing microplastic using 300 mL of aqueous lithium metatungstate (1.62 g cm^3^) to float out the microplastic particles from the soil sediments. For inorganic matter removal, density separation was done by adding NaCl solution to both water and sediment samples.

Besides that, Jaafar et al.^[^ ^4^ ^]^ have highlighted some additional clean‐up methods during post‐ingestion of extracted microplastic, namely sieving, density separation and oil extraction protocol (OEP) during extraction of microplastic from the GIT. A slight modification for sieving has been done using the combinations of stainless‐steel sieves with mesh sizes of 500 and 63 mm. The inclusion of a 63 mm sieve was necessary to remove the coloration from the digestate while also catching any potential plastic fragments larger than 63 mm. According to the author findings, the proportion of efficiencies by sieving was 98.73% percent for GIT and 99.22% for gills.^[^ ^4^ ^]^ It has been demonstrated that sieving aids in the elimination of undigested organic and inorganic contaminants and has been widely used for sediment samples. OEP is a novel technique that uses the oleophilic qualities of plastics to extract oil.^[^ ^111^ ^]^ Since the oil will interfere with the FTIR spectrum of microplastics, the sample should be purified with 90% ethyl alcohol following extraction.^[^ ^112^ ^]^ However, OEP is rarely used.

## 5 Methods for Microplastic Remediation

**Figure 3:**
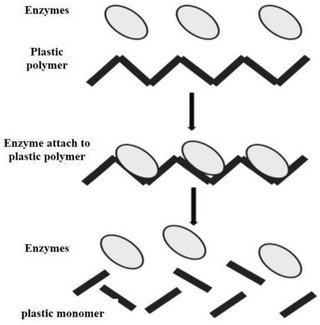
General mechanism of degrading microplastics into monomers through enzymatic process. Reproduced with permission.^[^ ^113^ ^]^ Copyright 2021, Springer Nature.

Remediation strategies are essential to eliminate the hazardous microplastic in the environment wherein removal technologies seem to be a more workable route. Exploiting microbes is one of the encouraging approaches for the remediation of microplastic. Microbes are utilized for the degradation of microplastic and called biodegradation process. Microorganisms such as algae, fungi, and bacteria have sparked scientists' interest as a potential treatment for microplastics. Basically, the enzymatic processes performed by microorganisms are intimately related to the breakdown of microplastics. Microorganism degrading enzymes can selectively target the polymer structure of microplastics and degrade them into monomers, which then can be utilized as a carbon source in the microorganism energy production cycle as shown in **Figure** **3**.^[^ ^113^ ^]^

However, not all enzymes are able to degrade microplastics. Enzyme including hydrolases (lipases, carboxylesterases, cutinases, and proteases) were reported to be accountable in altering microplastic polymer surfaces prone to deterioration via surface modification mechanisms and being called a surface modifying enzyme.^[^ ^114^ ^]^ This enzyme only improves the hydrophilicity of the microplastic surface while not degrading the microplastic's building blocks. Several enzymes have been identified as having the ability to breakdown polymers into monomers including oxidases, amidases, laccases, hydrolases, and peroxidases.^[^ ^115^ ^]^ **Table** **2** tabulates several degrading enzymes for the specific types of microplastic polymer. Meanwhile, **Table** **3** tabulates the methods reported by several countries including Malaysia to eliminate hazardous microplastic. Auta et al.^[^ ^116^ ^]^ have proposed biodegradation of microplastic using bacterial isolated from mangrove sediment in Peninsular Malaysia to reduce the environmental effect of microplastics. The result showed a positive impact when some of the bacterial used could reduce the weight loss of the investigated microplastic after 40 days. It is found that two isolates bacterials of *Bacillus cereus* and *Bacillus gottheilii* were able to degrade the microplastic materials of PE, PET, PP, and PS. *B. gottheilii* had a better capability to breakdown a wide range of microplastics than *B. cereus*, indicating that they had the potential to disintegrate when exposed to PP.

**Table 2:** Degrading enzymes for the specific type of microplastic polymers. Reproduced with permission.^[^ ^113^ ^]^ Copyright 2021, Springer Nature

Microplastic polymer	Degrading enzyme	Degradation mechanism
PE	– Laccase and alkane hydrolase	– Depolymerization followed by mineralization
PET	– PET degrading enzyme (PETase) and Secondary enzyme (MHETase)	– Abiotic and biotic degradation.
PS	– Styrene monooxygenase, styrene oxide isomerase, and phenylacetaldehyde dehydrogenase,	– Mineralization
PP	– Insufficient information	– No data reported.

**Table 3:** Summary on effective methods that might be suitable for microplastic removal in Malaysia

Method	Efficiency	Type of plastic	Advantages	Origin	References
Biodegradation	Degradation microplastic 40 days	PE, PET, PP, PS	– can reduce the use of chemical treatment	Malaysia	[116]
Adsorption	> 99%	PET	– Novel synthesized adsorbent using oil as carriers without stabilizing agents	Malaysia	[99]
Separation/ degradation	≥90% separation and ≥50% degradation	Various microplastics	– Highly efficient.‐Can be operated in WWTPs in various stages including primary, secondary, and tertiary	Canada	[117]
Coagulation	92 to 91.45%	PE, PET	– a magnetic coagulant containing of magnesium hydroxide (MgOH)/ Iron oxide (Fe_3_O_4_‐)Aging time of coagulant has no significant influence on removal efficiency.‐Coagulant aids enhanced the effect of adsorption and sweep flocculation.	China	[119, 120]
Electrocoagulation	96.5%	PE	– low operational costdiminishing both COD and thermotolerant coliforms	China	[122]
	>90%	PE, PMMA, PP and CA	– Removal efficiency increased with enhancement of electrolyte concentration and applied voltage density	China	[123]
Convection by solar energy	Utilize solar energy effectively	Any nano/microplastic	– Can remove plastic with free sunlight without chemicals or filters – Not require extra energy	China	[129]
Thermodegradation and biodegradation	Up to 98% carbon conversion for thermodegradation process	PS, PET, PP	– Biodegradation creates no secondary pollutants produced and potential for a large‐scale industrial level using a reactor system‐Thermodegradation can generate fuels, hence solving the problem relating to the energy security.	China	[130]
Bioremediation	‐Promising technique	Any nano/microplastic	– Bioremediation become effective in WWTPs the presence of higher eukaryotes (fungi).	Spain	[131]
Air flotation	69‐85%	PE, PVC, PES PE, PVC, PES	– pH of solution, size, and type of microplastic are the main factors influencing the removal efficiency of microplastic.	China	[132]
Low pressure membrane process	>90%		– Both ultrafiltration (UF) and microfiltration (MF) provide low energy requirements, compact plan size, simplicity, and scalability are just a few of the benefits of these processes		

Individual isolates may differ in metabolic rate, polymer uptake mechanism, and associated genetic modification, which could explain the discrepancies in response to diverse microplastics. *B. gottheilii* is a broad‐spectrum bacterium with affinity for all abovementioned types of microplastics studied. This bacterium can destroy a variety of plastics. Such behavior proved that the degradation of microplastic has practically occurred, hence appearing as a novel approach of green biodegradation for mitigation of microplastic in the environment. Also, this study can reduce the use of chemical treatment, which is not an environmentally friendly method. However, although bacteria species capable of degrading PP were mentioned in the study, no enzyme was identified as part of the degradation mechanism. Other research has been found in Malaysia wherein Hamzah et al.^[^ ^99^ ^]^ has modified the novel synthesized nano ferrofluid as an adsorbent using several types of oil as carriers without stabilizing agents or surfactants for microplastic removal via adsorption technique. The prepared adsorbent was effective in eliminating microplastic from laundry wastewater wherein 99 and 64% of microplastic were successfully removed from simulated water media and actual laundry wastewater, respectively.

On the other hand, Estahbanati et al.^[^ ^117^ ^]^ reported that the combination of both separation and degradation processes are amongst the promising approaches to treat microplastic in wastewater treatment plants (WWTPs). Separation process includes filtration, coagulation, flocculation, electrocoagulation, air flotation etc. Meanwhile degradation techniques including catalytic degradation, photocatalytic degradation, and electro oxidation. As reported, at least 90% of microplastics could be removed using the separation techniques. The efficiency of using degradation procedures is lower than that of separation processes, and it can be as low as 50% (catalytic degradation). This combination technique is suitable for the treatment of microplastic at WWTPs. Normally, during microplastic treatment, they undergo primary, secondary, and sometimes tertiary stages in WWTPs. According to prior research findings, secondary treatment commonly eradicates microplastics larger than 20 µm.^[^ ^118^ ^]^ Hence, the tertiary stage should be capable of successfully eliminating microplastics with a diameter of less than 20 µm. It can be inferred that multiple secondary and tertiary treatment techniques must be developed to treat diverse types and concentrations of microplastics.

Besides, coagulation is one of the conventional methods that can be used for microplastic treatment as well as can be modified for their best performance. A study has reported the removal of 92% PE from wastewater via a modified coagulation method using magnetic MgOH and polyacrylamide (PAM).^[^ ^119^ ^]^ This method can eliminate the microplastic in a wide range of pH from 5–9, as well as an aging time of the coagulant insignificantly affect the microplastic removal. Another research by Zhang et al.^[^ ^120^ ^]^ also showed an improvement in the coagulation method when using coagulant aids such as anionic PAM, sodium alginate (SA), and activated silicic. The result exhibits that ≈91.45% of PET has been successfully extracted when using PAC with a high dose of PAM. This coagulant enhanced the effect of adsorption as well as sweeping the flocculation. Meanwhile, electrocoagulation, an electrochemical method, provides a low‐cost three‐stage wastewater treatment process that does not rely on the chemicals or microbes used in standard activated sludge processes.^[^ ^121^ ^]^ This electrochemical technique has been utilized for contaminants removal from water because of its environmental compatibility, cheap, energy efficiency, sludge minimization, ease of automation, and cost‐effectiveness. Electrocoagulation is a complex process in which an electric field causes metal electrodes to create cations forming micro coagulant. When colliding with flocks, they are combined with suspended particles and sink together. Then, the coagulant forms a sludge layer to retain suspended solid particles.^[^ ^122^ ^]^About 96.5% of microplastic was effectively removed from natural wastewater using electrocoagulation at low operational cost as well as diminishing both chemical oxygen demand (COD) and thermotolerant coliforms.^[^ ^122^ ^]^ Shen et al.^[^ ^123^ ^]^ also reported the removal of various microplastics such as PE, PMMA, PP and CA with high extraction efficiency >90% using electrocoagulation. This removal efficiency increased with augmentation of electrolyte concentration and applied voltage density.

Then, adsorption technique also has been a great potential in eliminating microplastic using various types of adsorbent. Some studies also highlight the use of antibiotics on microplastics adsorption. For instance, Yu et al.^[^ ^124^ ^]^ have reported the use of a typical antibiotic of tetracycline (TC) as adsorbent tested on several types of microplastics such as PE, PS, PVC, and PET of various sizes. Isotherm fitting revealed that PE had the highest maximum adsorption capacity and coefficient, as well as the strongest adsorption capacity, of the three microplastics studied: PE, PS, and PVC. Wang et al.^[^ ^125^ ^]^ also proposed an effective magnesium/zinc (Mg/Zn) modified magnetic biochar adsorbents for the removal of microplastics. The removal efficiencies of magnetic biochar (MBC), Mg modified magnetic biochar (Mg‐MBC), and Zn modified magnetic biochar (Zn‐MBC) for PS microspheres (1 µm) in aqueous solution were 94.81%, 98.75%, and 99.46%, respectively. Electrostatic contact and chemical bonding interaction between microplastics and biochar are believed to induce the adsorption process. The adsorption capacity was maintained after thermal regeneration wherein MBC (95.02%), Mg‐MBC (94.60%), and Zn‐MBC (95.79%) have shown good microplastic removal effectiveness even after five adsorption‐pyrolysis cycles.

Meanwhile, microbubble technique is a new insight that can significantly increase flotation performance.^[^ ^126^ ^]^ Factors such as diameter of the microbubble, staying time, intense density, and extreme interfacial area enhance the quality of flotation process by increasing the collision effect on bubbles and particles. This technique was widely utilized to remove extremely small particles from the water system including microplastic.^[^ ^127^, ^128^ ^]^ To date, Wang et al.^[^ ^129^ ^]^ have proposed a new strategy for more sustainable microplastic removal, which is driven by solar energy. Throughout this method, sunlight was focused through a glass ball with high power density, inducing convection and forming a microbubble at the interface. During the convection process, the microplastic was driven into the microbubble without using any chemicals or filters. Additionally, this method is simple that can consume free sunlight without needing additional energy sources or causing secondary pollution. Not only that, Arpia et al.^[^ ^130^ ^]^ have reviewed that thermodegradation and biodegradation of microplastic appear as the sustainable routes for microplastic degradation. Biodegradation can be a profitable method since no secondary pollutants are produced and potentially be applied at a large‐scale industrial level using a reactor system. However, this method needs physicochemical pretreatment to crumble polymers into the monomers form. Concurrently, the thermodegradation of microplastic can generate fuels, hence capable of solving the problem relating to energy security. Additionally, research is required to incorporate this technique with the simulation program to optimize this approach.

According to Masia et al.,^[^ ^131^ ^]^ bioremediation also has been identified as a promising method for microplastic removal from WWTPs. However, the possibility of microplastic bioremediation in the WWTPs become effective in the presence of the higher aquatic eukaryotes (fungi), which have low dispersion rates and are manageable. Amongst the characteristics of higher eukaryotes that are important to their usage in bioremediation including the capture and elimination of microplastic should be high and they should not be released to the environment. Second, species should only be used within their native range, as geographical transfers must be avoided for the sake of biodiversity conservation. Besides, species having a wider distribution, as well as easier control and management, would be preferable. As using a species for WWTP treatments that being cultivated inside or near treatment plants, containment techniques to prevent them from releasing to the environment must be effective. Other potential marine animals are seagrasses and macrophytes also appear to be good possibilities, with some measures to keep species controlled. On the other hand, Pramanik et al.^[^ ^132^ ^]^ have examined the air flotation, and membrane process as a proof‐of‐concept study for the removal of microplastic. Fundamentally, flotation technique has been widely employed in municipal wastewater treatment to remove ultra‐fine and particulate, colloidal and greasy emulsions. This process suspends particles to the liquid surface to remove them from the liquid. Bubble creation, bubble attachment, and solids separation are the three essential processes in this method. It has a number of advantages over traditional filtration and precipitation procedures, including better water quality, faster startup, and a higher operation rate.^[^ ^133^ ^]^ According to Pramanik et al.,^[^ ^132^ ^]^ the type of microplastics had a substantial impact on the removal efficiency since different microplastic have different levels of floatability. Such behavior is due to the different density of microplastic. It was found that an air flotation was discovered to be capable of removing 69‐8%of PE, PVC, and PES. The effectiveness of the air flotation system in removing these plastic particles was determined by the pH of the solution as well as size and type of the microplastics.

Besides, both MF and UF are low pressure membrane separation categorized in tertiary treatment process that can control microplastic pollution in effluent. Small particles or components from various sources of wastewater can be rejected throughout these two processes.^[^ ^134^ ^]^ Advantageously, low energy requirements, compact plan size, simplicity, and scalability are just a few of the benefits of these processes. The size of the particles was discovered to have a substantial impact on their filterability. According to Pramanik et al.,^[^ ^132^ ^]^ the pores of UF membranes were substantially smaller than those of the MF membranes, there was a higher removal of microplastics by UF than MF membrane. Results revealed that UF and MF could reduce microplastics up to 96% and 91%, respectively.

## 6 Challenges and Perspective for Plastic Pollution Management in Malaysia

Plastics undergo fragmentation into microplastics and provide hazardous threats in all life forms, including human beings. To date, numerous solutions are proposed to treat microplastic from the environment which can be categorized into containment, mitigation, and remediation.^[^ ^130^ ^]^

### 6.1 Containment Approach

The containment approach mainly focuses on recycling and proper landfill management.^[^ ^135^ ^]^ Chen et al.^[^ ^40^ ^]^ have proposed a model for plastic waste management in Malaysia using a concept combining a circular economy introduced by Kirchherr et al.^[^ ^136^ ^]^ and an integrated solid waste management hierarchy. Amongst recommendations identified for Malaysia include a comprehensive data evaluation for current waste disposal methods and recycling.

**Figure 4:**
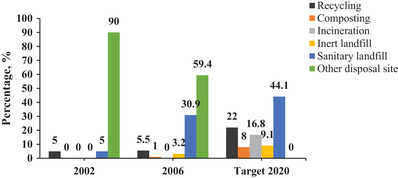
Treatment methods practiced for waste management in Malaysia. Reproduced with permission.^[^ ^137^, ^140^ ^]^ Copyright 2009, Elsevier, Copyright 2013, Penerbit UTM.

**Figure** **4** shows few treatment methods being practiced for plastic waste management in Malaysia. Conventional landfilling is the most superior method used in Malaysia due to the lowest cost, and normally, sites used are open dumping areas.^[^ ^137^ ^]^ Unfortunately, these areas have led to a severe impact on the environment, such as contaminated surface water, groundwater and soil since they are directly or indirectly in contact with this waste. Also, once landfilled, this waste undergoes a series of physicochemical and biological revolutions, thus contaminating wastewater with leachate. The leachate composition differs among landfills, predominantly affected by type of waste buried, degradation phase, climate circumstances, features of landfill sites, socioeconomic factors, and landfill technology.^[^ ^138^ ^]^ This disposal method greatly contaminates the river water with leachate.^[^ ^139^ ^]^

Not only that, compared to the urban areas, but mostly waste collection in the rural areas is also not fully covered. Such a situation causes the waste to be omnipresent in the rural area, leading to serious environmental issues such as flooding, disease, etc. Several efforts have been performed to improve the landfill system. According to Samsudin and Don,^[^ ^140^ ^]^ higher tipping fees should be charged to industries that sending their wastes to landfill sites for disposal to increase the motivation on waste minimization and sustain good landfill practices. Besides, compostable, biodegradable plastics can be grouped with the food and organic wastes before being sent to composting facilities as a green substitute for chemical fertilizers.^[^ ^40^ ^]^ Also, to decrease the environmental problems due to open dumping areas, engineered landfills and sanitary landfills should be provided in Malaysia, which comes with a full set of measures for methane gas control, leachate treatment, and aftercare plan for landfills.^[^ ^40^, ^140^ ^]^

**Figure 5:**
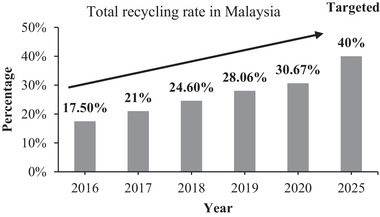
Total recycling rate of waste in Malaysia. Reproduced with permission.^[^ ^148^ ^]^ Copyright 2020, Sinar Harian.

As observed in Figure 4, there was no waste incineration practiced until 2006. According to Fazeli et al.,^[^ ^141^ ^]^ the earlier usage of unsanitary landfills might effectively reduce incineration and concurrently generate energy comparable to the sum of fossil fuels. On the other hand, recycling, composting, incineration, inert landfill, and sanitary landfill were targeted with high percentages in 2020, which are 22,8,16.8, 9.1 and 44.1%, respectively. As shown in **Figure** **5**, the total recycling rate in Malaysia keep increasing annually and reached above the target of 30.67% in 2020. Through this survey, it can be revealed that most Malaysians seem to be in lacking space at home for recycled materials. However, this percentage is still lower compared to the other developed countries such as Germany, United Kingdom, and Singapore, with their recycling rate above 40%. A recycling rate of 40% is targeted by Scheduled waste cooperation (SWCorp) to be achieved in Malaysia by 2025.^[^ ^50^ ^]^

Malaysia suffers a limitation in terms of recycling plastic, which can recycle certain categories of plastic, namely PET, high‐density polyethylene (HDPE) and polypropylene (PP).^[^ ^142^ ^]^ Referring to the National Solid Waste Management Department, mechanical recycling is a common method to reproduce the newly formed plastic while still maintaining its initial chemical composition.^[^ ^143^ ^]^ Although we can recycle the abovementioned type of plastic, the local recycling industry only focuses on easily retrievable and high‐value plastics such as PET.^[^ ^144^ ^]^ The low‐grade plastic used for food packaging is seldom recycled, hence causing our landfills highly filled with this low quality plastic.

Wahab et al.^[^ ^145^ ^]^ has carried out case studies associating with the recycling trend of a recycling company in Malaysia. It is found that almost 80% of local manufacturing companies consumed recycled plastic materials due to cost savings, with 20% of these companies performed in‐house recycling. The recycled materials were obtained from various sources, namely industrial scrap, dumping sites, local producers and imported sources. However, according to MPMA,^[^ ^34^ ^]^ mostly the recycling companies in Malaysia prefer imported recycled plastic over the local sources since they provide lower prices, large volume, homogeneous and guaranteed supply. In Malaysia, incineration of waste is not highly recommended since the cost of waste conversion to energy is not economical enough due to the high‐level moisture in waste in Malaysia.^[^ ^141^ ^]^ A study has proposed a combination of waste with the agricultural residue biomass during incineration for higher efficiency.^[^ ^146^ ^]^ Besides, not all plastic types are applicable for incineration, such as polyvinyl chloride and polystyrene, since they can release toxic gas during the process.^[^ ^147^ ^]^

According to Chen et al.,^[^ ^40^ ^]^ these types of plastic should be separated appropriately using an automatic sorting plant or manual sorting using a workforce that can be costly. It is advised that the government should focus on waste recovery via recycling methods while reducing the environmental problem caused by incineration plants. Even though the containment strategy has a positive impact on reducing plastic contamination in the environment, it is still implausible to be the best solution.

### 6.2 Mitigation Strategy

Mitigation strategy focuses on the practice of regulations and legislative measures. The mitigation's goal is to enhance people's awareness of the harmful threat of microplastic. Some countries have banned plastic bags, such as New Zealand (since 2019), Thailand (since 2020) and India (next 2022).^[^ ^144^ ^]^ For sustainable development, the Malaysian government has introduced a plan on “Malaysia's Roadmap to Zero Single‐Use Plastics” starting from 2018 to 2030.^[^ ^2^ ^]^

Earlier in 2011, the Malaysian government has organized a program “No‐Plastic Bag Day” campaign each Saturday to diminish the ecological pollution from used plastic bags using a “market” as an instrument to prevent consumers from using plastic bags for items purchased. A levy of MYR0.20 was charged for each new plastic bag demanded by customers during the program. From the findings, it is observed that the participation of consumers in the abovementioned program is moderate, with a 52.3% reduction, and the remaining 47.7% of consumers are willing to pay the charges or tax for plastic bags.^[^ ^149^ ^]^

Data from ministry of energy, science, technology, environment and climate change (MESTECC)^[^ ^144^ ^]^ showed that other few states in Malaysia, namely Selangor, Kedah, Pulau Pinang and Pahang, have organized a “No Plastic Bags” campaign to support the country's goal of having zero single‐use plastics in future. This effort will slowly create awareness among Malaysians if implemented effectively compared with the countries that show a positive impact when the reduction of plastic used higher than 50%, such as Denmark, Portugal and England.^[^ ^150^, ^151^, ^152^ ^]^

According to Chen et al.,^[^ ^40^ ^]^ the Malaysian government requires tougher implementation of regulations and extra hard work to enhance environmental awareness and commitment among Malaysians. In 2014, MPMA promoted the “Reduce, Reuse and Recycle (3Rs)” program to enhance community consciousness of plastic recycling. It is found that only 26% of participation for reduction activities come from households, 20% in reuse practice, while 29% take part in separation at source.^[^ ^153^ ^]^

Ting et al.^[^ ^154^ ^]^ have applied the theory of planned behavior (TPB) in exploring the reasons influencing Malaysians' application of the reducing, reuse and recycle (3Rs) idea in plastic usage. This theory offers a well understanding of the correlation among the determinants and the 3Rs' target on plastic usage behavior intention. Findings revealed an important and optimistic association among attitude, habit, assisting situations and 3Rs behavior. To effectively implement the 3Rs concept among Malaysians, the recycling facilities in the residential areas must be increased. Supporting rules from the government side are highly needed to change Malaysian's behavior.

Mei et al.^[^ ^155^ ^]^ has carried out a case study on the environmental awareness among Malaysians toward four categories: water pollution, air pollution, waste management, and climate change. It is found that most Malaysians are aware of water and air pollution, followed by solid waste management and climate change. Also, Zen et al.^[^ ^156^ ^]^ has conducted a case study associating with the profiling background and revealed that most of the household recyclers in Kuala Lumpur, Malaysia, come from well‐educated people with higher income earners, own houses and have excellent knowledge of recycling. Conversely, the non‐recyclers have a lower education background, earn lower incomes, tenants in one‐storey houses with little knowledge of recycling. A similar observation was reported by Aminrad et al.,^[^ ^157^ ^]^ who reported that the higher education level makes students better understand the bad effect of plastic consumption on environmental pollution. However, these findings contradict Hassan et al.,^[^ ^158^ ^]^ who indicated that even though the educated person is knowledgeable, it does not increase their positive manners toward environmental concerns.

Additionally, it is also found that female students possess high positive behaviors compared to male students to reduce plastic consumption. This is in line with the other studies that claimed that women possess pro‐environmental behavior compared to men due to diverse patterns of socialization.^[^ ^159^, ^160^ ^]^ On the contrary, a study by Pakpour et al.^[^ ^161^ ^]^ claimed that male students are more concerned about the environment than female students. However, a study revealed that there was an insignificant difference in the genders factor when concerning the environmental issues.^[^ ^157^ ^]^ Moh and Manaf^[^ ^162^ ^]^ have surveyed awareness of waste separation among Malaysian households in Johor, Malaysia. It is found that 36.46% have heard of it and support this while 36.54% of them just know about this matter only through this survey. Those who just heard about this should be turned into committed recyclers using awareness campaigns. Through this survey, it can be revealed that most Malaysians seem to lack space at home for recycled materials. Omran et al.^[^ ^163^ ^]^ mentioned that circumstances of the physical environments are also one of the critical factors in recycling behavior.

According to Chen et al.,^[^ ^40^ ^]^ Malaysia should have a strict implementation in the waste separation for the household group. Among them are keeping the residents aware of the regulations and opportunities for recycling, integrating housing developers into the governance structure, proper signage in housing areas, providing suitable disposal bins, and executing non‐compliance with penalties. This strategy also helps reduce microplastic pollution but is ineffective for microplastics that come from properly disposed of waste.^[^ ^164^ ^]^

### 6.3 Remediation Strategy

In terms of remediation, microplastics are currently not included in the treatment scope of sewage treatment plants, and research into the treatment efficiency of microplastics is still in its early stages. Although microplastics can be removed in a sewage treatment plant using existing treatment methods, some microplastics can still bypass the treatment plant and reach the aquatic environment. As observed, there are not many approaches reported by Malaysian researchers regarding microplastic remediation. In terms of the wastewater treatment plant, tertiary treatment needs to be improved to reduce the amount of microplastics in the final effluent being released to the environment. Estahbanati et al.^[^ ^117^ ^]^ reported that the combination of both separation and degradation processes are amongst the promising approaches to treat microplastic in WWTPs for the secondary and tertiary stages of treatment. The main challenge for microplastic pollution is improving tertiary wastewater treatment, particularly the filtering process. Ozone, quicksand filtration, reverse osmosis, dissolved air flotation, and membrane bioreactors are some of the wastewater treatment solutions offered.^[^ ^165^, ^166^ ^]^ For sludge production, oxidation and decomposition treatments have been recommended to free trapped microplastics.^[^ ^165^ ^]^

## 7 Green Strategies on Microplastic Reduction

Several strategies can be conducted to achieve a green environment with lower amount of microplastics waste. Initially, measures focusing on the reduction of plastic waste at the industrial level are necessary. For instance, eco‐designing schemes can be applied to change the existing method plastics being manufactured with the aim of reduction or prevention and reuse that involves a delicate mix of rules and incentives. Other alternatives are the development of plastics that are devoid of harmful compounds, use of alternative materials, or the expansion of long‐lasting plastics.^[^ ^167^, ^168^, ^169^ ^]^ Meanwhile, prohibiting the use of certain single‐use plastics such as bags, food packaging, bottles, and containers that are only used once before being discarded can be applied by several governments around the world to reduce the plastic pollution in the ocean.^[^ ^170^ ^]^ Also, recycled plastic market need to be supported with several initiatives such as imposing taxes on the usage of single plastics, create awareness regarding the environmental benefits of recycled plastics as well as incentivizing the manufacture of recycled plastics.^[^ ^171^ ^]^ Besides that, the introduction of new bioplastic materials and biodegradable plastic that meet the same requirements with the existing bioplastics with a lower environmental impact is highly needed.^[^ ^172^ ^]^ The bioplastic made from biomass resources is capable of boosting resource efficiency using biomass material, conserving fossil‐fuel resources, reducing greenhouse gas emissions and carbon footprint, as well as minimizing the total production costs.^[^ ^172^, ^173^ ^]^ These bioplastics are commonly used for pharmaceutical components, medication capsules, household appliance, and agricultural products.^[^ ^173^ ^]^

## 8 Conclusions

An environmental problem caused by microplastics contamination is unceasingly occurred and might put Malaysia in a critical state without proper countermeasures. Microplastic pollution have been reported in Malaysia via ingestion of benthic organisms, surface water and sedimentation. However, the number of research efforts seemed to be relatively high toward marine, while freshwater, lakes, and reservoirs have received much less research attention. Further research should focus on the freshwater and terrestrial ecosystems to provide better insight on the sources, abundance, and distribution of microplastics. Even though the ingestion of microplastics has been reported in a variety of animals taken from aquatic environments, there is little information in the literature on the intake of microplastics through trophic interactions, and the long‐term impacts of trophic transfer are mostly unclear. Therefore, field and laboratory research must be done to comprehend the effects of microplastics transfer at trophic levels in Malaysia. The toxicity of microplastics to aquatic organisms is unknown, however prior research has shown that the abundance of microplastics in aquatic organisms is not high enough to have a hazardous effect. It is possible that the additives that microplastics contain constitute a bigger threat. Considering the impacts and interactions of microplastics and chemicals, it is necessary to put an emphasis on addressing the ecotoxicological risks of microplastics in future. Besides, more study is needed to understand how microplastics interact with metals and organic contaminants for a better estimation of the risk posed by microplastics. It is crucial to have a deeper understanding of microplastic pollution in the aquatic environment. On the other hand, research on the treatment of microplastic has been extremely limited in Malaysia. To avoid risks to human health, it is necessary to evaluate and enhance the treatment processes for eliminating microplastics from contaminated water. The current issue that must be solved is developing the technology to legislate contamination due to the complex mixture of various microplastic with other contaminants. In terms of knowledge, technology, and resources, initiatives to establish networks of scientists and carry out intense in‐country trainings or workshops can further contribute and can promote efficient approaches to develop the microplastic research in the future.

## Conflict of Interest

The authors declare no conflict of interest.
